# Plant functional traits are correlated with species persistence in the herb layer of old-growth beech forests

**DOI:** 10.1038/s41598-020-76289-7

**Published:** 2020-11-06

**Authors:** Giandiego Campetella, Stefano Chelli, Enrico Simonetti, Claudia Damiani, Sandor Bartha, Camilla Wellstein, Daniele Giorgini, Nicola Puletti, Ladislav Mucina, Marco Cervellini, Roberto Canullo

**Affiliations:** 1 Unit of Plant Diversity and Ecosystems Management, School of Biosciences and Veterinary Medicine, University of Camerino, Via Pontoni, 5, 62032 Camerino, Italy; 2 School of Biosciences and Veterinary Medicine, University of Camerino, Via Gentile III da Varano, 62032 Camerino, Italy; 3 Institute of Ecology and Botany, Centre for Ecological Research, 2163 Vácrátót, Hungary; 4 GINOP Sustainable Ecosystems Group, Centre for Ecological Research, Klebelsberg Kuno u. 3, 8237 Tihany, Hungary; 5 Faculty of Science and Technology, Free University of Bozen-Bolzano, Piazza Università 5, 39100 Bozen, Italy; 6 Council for Agricultural Research and Agricultural Economy Analysis, Forestry Research Centre, Viale Santa Margherita 80, 52100 Arezzo, Italy; 7 Harry Butler Institute, Murdoch University, 90 South Street, Murdoch/Perth, WA 6150 Australia; 8 Department of Geography and Environmental Studies, Stellenbosch University, Private Bag X1, Matieland, 7602 Stellenbosch South Africa; 9 Department of Biological, Geological, and Environmental Sciences, Alma Mater Studiorum, University of Bologna, 40126 Bologna, Italy

**Keywords:** Forest ecology, Population dynamics

## Abstract

This paper explores which traits are correlated with fine-scale (0.25 m^2^) species persistence patterns in the herb layer of old-growth forests. Four old-growth beech forests representing different climatic contexts (presence or absence of summer drought period) were selected along a north–south gradient in Italy. Eight surveys were conducted in each of the sites during the period spanning 1999–2011. We found that fine-scale species persistence was correlated with different sets of plant functional traits, depending on local ecological context. Seed mass was found to be as important for the fine-scale species persistence in the northern sites, while clonal and bud-bank traits were markedly correlated with the southern sites characterised by summer drought. Leaf traits appeared to correlate with species persistence in the drier and wetter sites. However, we found that different attributes, i.e. helomorphic vs scleromorphic leaves, were correlated to species persistence in the northernmost and southernmost sites, respectively. These differences appear to be dependent on local trait adaptation rather than plant phylogenetic history. Our findings suggest that the persistent species in the old-growth forests might adopt an acquisitive resource-use strategy (i.e. helomorphic leaves with high SLA) with higher seed mass in sites without summer drought, while under water-stressed conditions persistent species have a conservative resource-use strategy (i.e. scleromorphic leaves with low SLA) with an increased importance of clonal and resprouting ability.

## Introduction

Vegetation dynamics are complex phenomena involving many processes underpinning changes of vegetation patterns, such as directional succession of plant assemblages, cyclic patterns emerging as consequence of recurrent disturbance events^1^, or fluctuating population-dynamic trajectories ensuring community stability responding to, for instance, interannual variability of the weather^2^. While successional and cyclic dynamics have received much attention, this is less the case for fluctuations, especially in forest ecosystems^3–5^.

Recent studies of forest dynamics have considered the importance of clonal mobility (i.e. spatio-temporal patterns of plant individuals) for vegetation patterns^6^, examined temporal species turnover^7,8^, and explored the impact of forest management on forest floor vegetation^9^. While it has been strongly suggested that long-term studies should pursue multiple essential objectives to understand critical ecological mechanisms^10^, very few studies focusing on fine-scale species persistence have used long-term datasets covering at least a decade and different observation targets over time. Fine-scale observations are essential, as they can provide crucial information on the genesis and maintenance of diversity^11^ and seem fundamental for understanding large-scale spatial and temporal processes and their underpinning mechanisms^5,12^. For example, Nygaard & Ødegaard^4^ and Økland & Eilertsen^3^ found changes in species composition due to variation in soil pH and water availability in boreal forests. Økland^13^, who re-analysed permanent plots annually from 1988 to 1993 in boreal forests and related persistence to some clonal traits, emphasised the importance of clonal growth, ramet longevity and mobility for species persistence. However, more studies are needed, especially those employing traits related to various plant functions and different environmental and/or climatic contexts. It has been demonstrated that species dynamics strongly depend on the environmental context^8,14–16^. In seeking to understand the complexity of vegetation dynamics, a crucial factor in analysing is *species persistence*—the tendency of a species to maintain the spatial original position^12,13^. Likely, the patterns in the relationships between plant persistence and traits would change as an adaptive response, possibly dependent on locally differentiated plant communities (species composition) and plant traits abundance distribution.

The herb layer of old-growth forests (in this context, those older than 100 years) is characterised by ecological continuity as the relatively stable ecological conditions may persist for a long time. This continuity can activate environmental filters favouring the colonization and persistence of specialist species^17,18^. It has been demonstrated that well-adapted understory species can influence the long-term stability of the ecosystem^19^; they also affect the microbiological processes and nutrient cycling of the organic layer^19^. Thus, these species are widely considered to be of high conservation value^14,18,20^. Therefore, it is vital focus on a question asking which functional traits are essential for the persistence of these species. Plant functional traits are a very useful tool in understanding mechanisms that shape species patterns within ecosystems^21^. In detail, Weiher et al.^22^ identified seed, leaf, and clonal traits as those traits informing about the significant challenges of plant persistence. The seed and leaf traits provide information about resource acquisition and use strategies (e.g. Leaf–Height–Seed scheme^23^; Leaf Economic Spectrum^24^). Nevertheless, other key plant functions related to different ecological dimensions, such as space occupancy and recovery after damage, remained largely neglected^22^. Traits that can effectively capture these understudied functions are those associated with clonality (traits linked to vegetative reproduction and clonal spreading) and bud bank (traits related to the occurrence of stem- and root-derived buds)^25^.

Beech forest specialist species are usually clonal species^15^, characterised by larger specific leaf area (SLA), higher multiplication rates, and higher seed mass^14^. However, the crucial traits related to their persistence are still unknown, especially for cold temperate and Mediterranean forests. Further, it is not clear if the sharing of similar traits and the preference for the same (stable and shady) environment is better reflected by community phylogenetics or by functional adaptations^26^.

**Figure 1:**
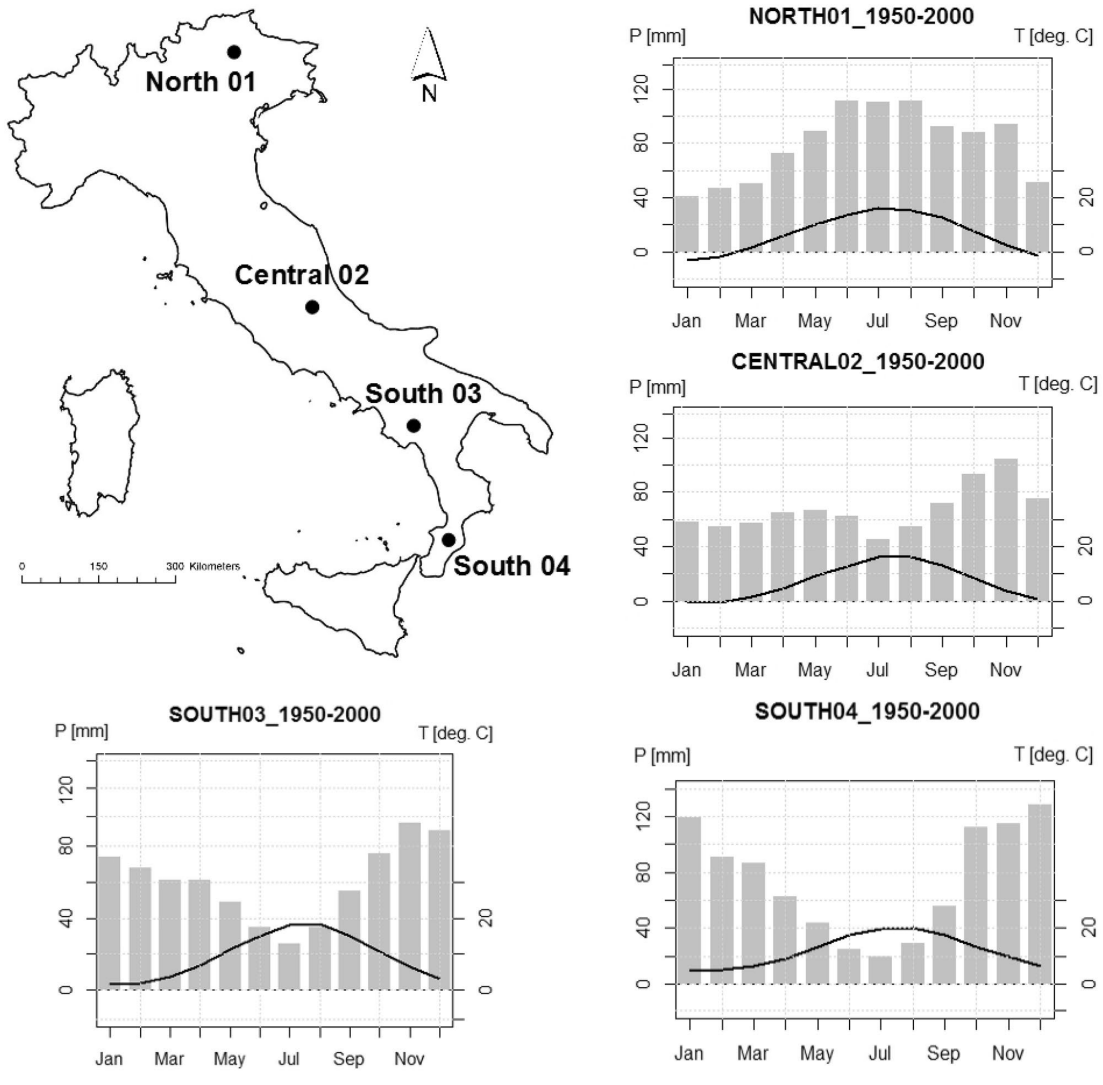
Location of the four sites and Bagnouls-Gaussen climate diagrams (based on WorldClim 50 years meteorological data^51^).

A unique opportunity to examine the traits-driven species persistence is provided by the Italian Forest Ecosystem Monitoring Network. This Network involves Permanent Monitoring Plots (hereafter, ‘sites’), where the ground vegetation assessment was performed repeatedly at a fine scale (0.25 m^2^) for longer than a decade. We selected four mature old-growth beech (*Fagus sylvatica* L.) forests representing different climatic contexts (presence or absence of summer drought period; Fig. 1) along a north–south gradient in Italy. We used the data of the monitoring of those forests to explore the trait-based mechanisms of species persistence in forest understory.

Firstly, we verified whether the local ecological conditions of each site were so profoundly site-specific that they significantly differentiated not only the understory species composition but also the plant traits pools and relative abundance distribution. Secondly, we assessed if the local functional patterns were correlated with phylogenetic signals. Then, we devised an index of species persistence by using both presence/absence and abundance data. This combined approach was designed to reveal the potential different behaviour of dominant with respect to subordinate species. As the final step, we ascertained which traits were correlated with fine-scale species persistence, and hence formulated the following hypotheses:

### H1:

Seed mass is related to seedling survival and is usually larger in plants in old-growth forests^9,27^ characterised by closed canopies and shaded understories. Therefore, *we hypothesise that larger seed mass would favour species persistence across all four old-growth beech forest stands.*

### H2:

Among the leaf traits, SLA is related to water-use efficiency, photosynthetic capacity, and relative growth rate that, in turn, is under control of water, light, and nutrient availability^28,29^. Leaf anatomy^30^ is related to both water and nutrient conservation and prevention of mechanical damage^31^. Therefore, *we expect lower SLA and presence of scleromorphic leaves to favour species persistence in forests experiencing marked summer drought.*

### H3:

Clonal traits related to mobility support plant persistence in habitats with more stress-inducing (drought) conditions, increasing the fine-scale spatial resource acquisition by increasing the foraging ability^11,32,33^. Further, bud bank traits related to regeneration reflect plants ability to re-sprout after disturbance^14,15,34^. Based on this rationale, *we hypothesise that clonal and bud-bank traits would favour species persistence in forests more impacted by summer drought.*

## Results

### Differences in species composition and trait abundance among the sites

PERMANOVA revealed that at the beginning of the monitoring the four sites differed significantly in terms of species composition and traits abundance (species composition *R*^2^ = 0.35, *p* = 0.001, df = 3; traits abundance *R*^2^ = 0.48, *p* = 0.001, df = 3).

### Relationship between functional and phylogenetic patterns

The site-level correlations between functional and phylogenetic diversity were low and non-significant (North01, rho = 0.062, *p* = 0.539; Central02, rho = 0.045, *p* = 0.844; South03, rho = 0.114, *p* = 0.271; South04, rho = 0.117, *p* = 0.252).

### Plant functional traits and species persistence

**Figure 2:**
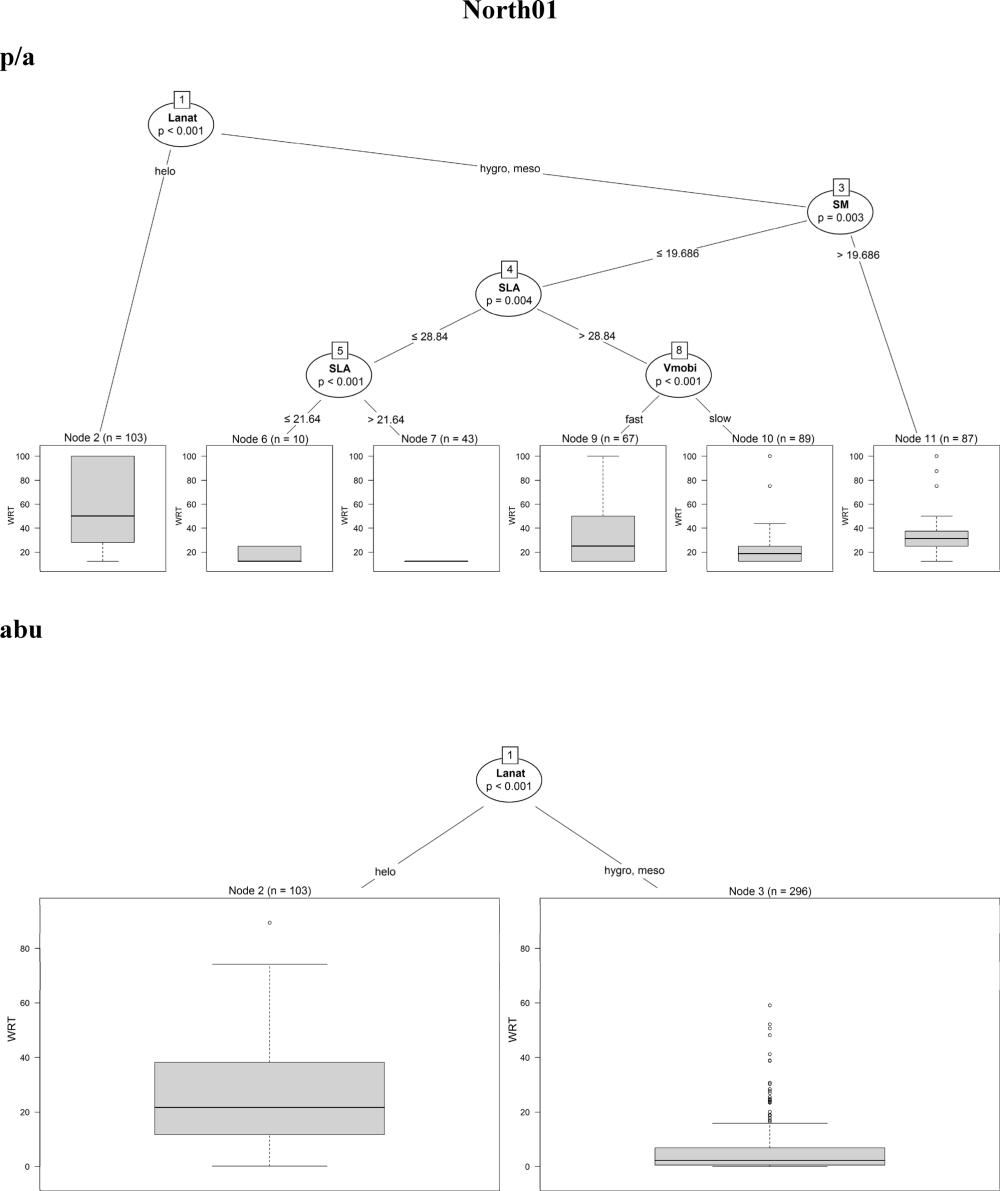

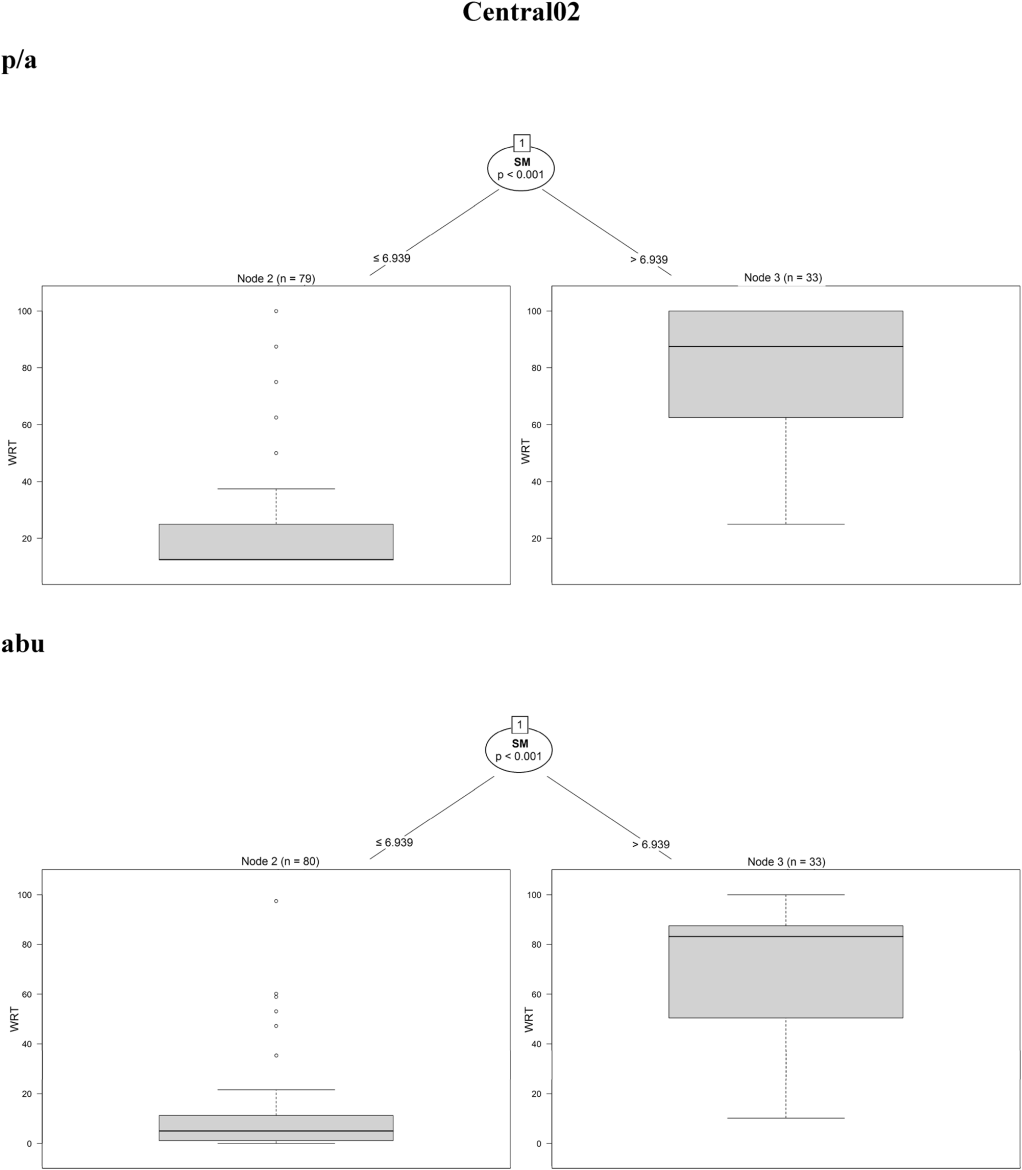

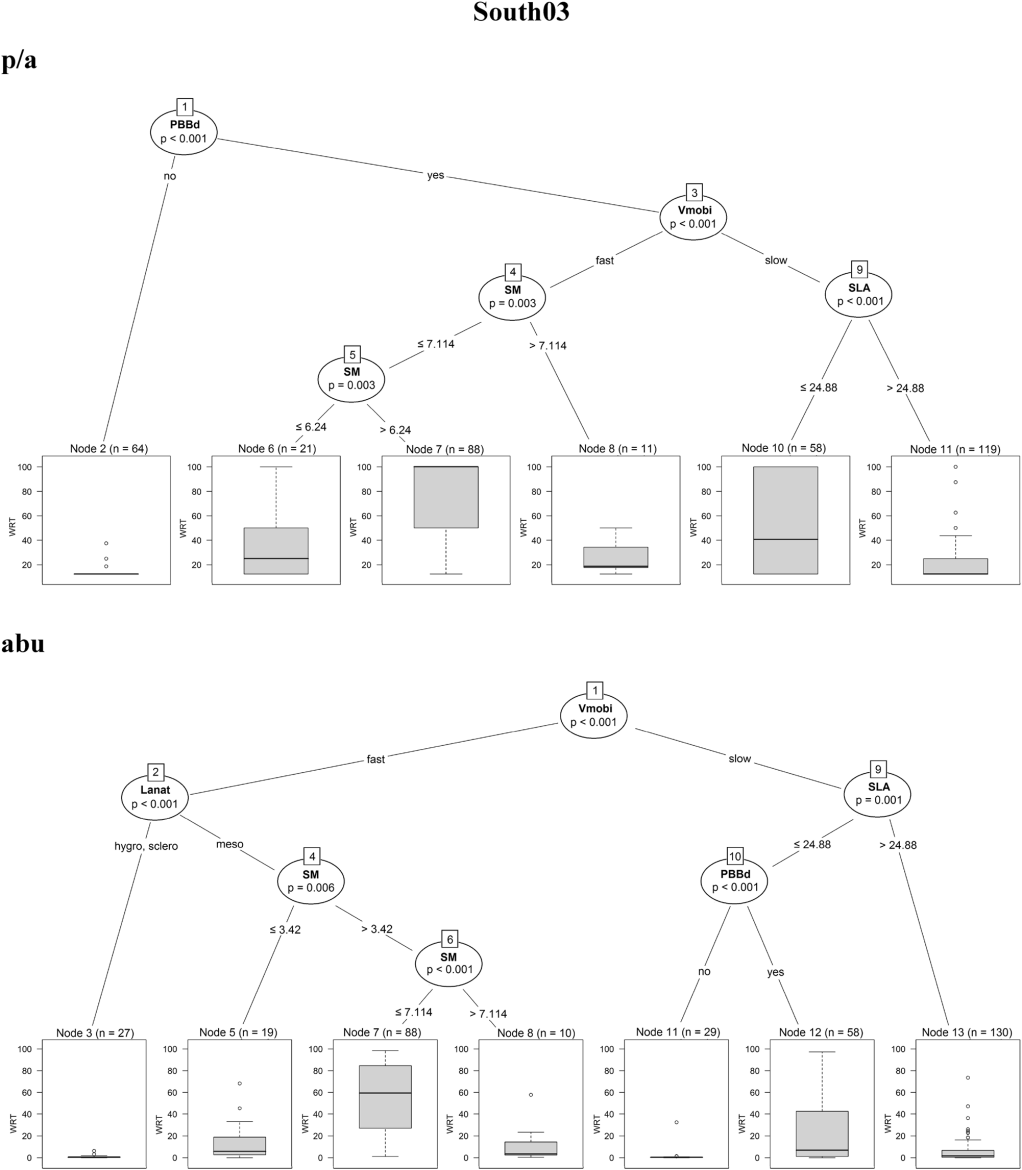

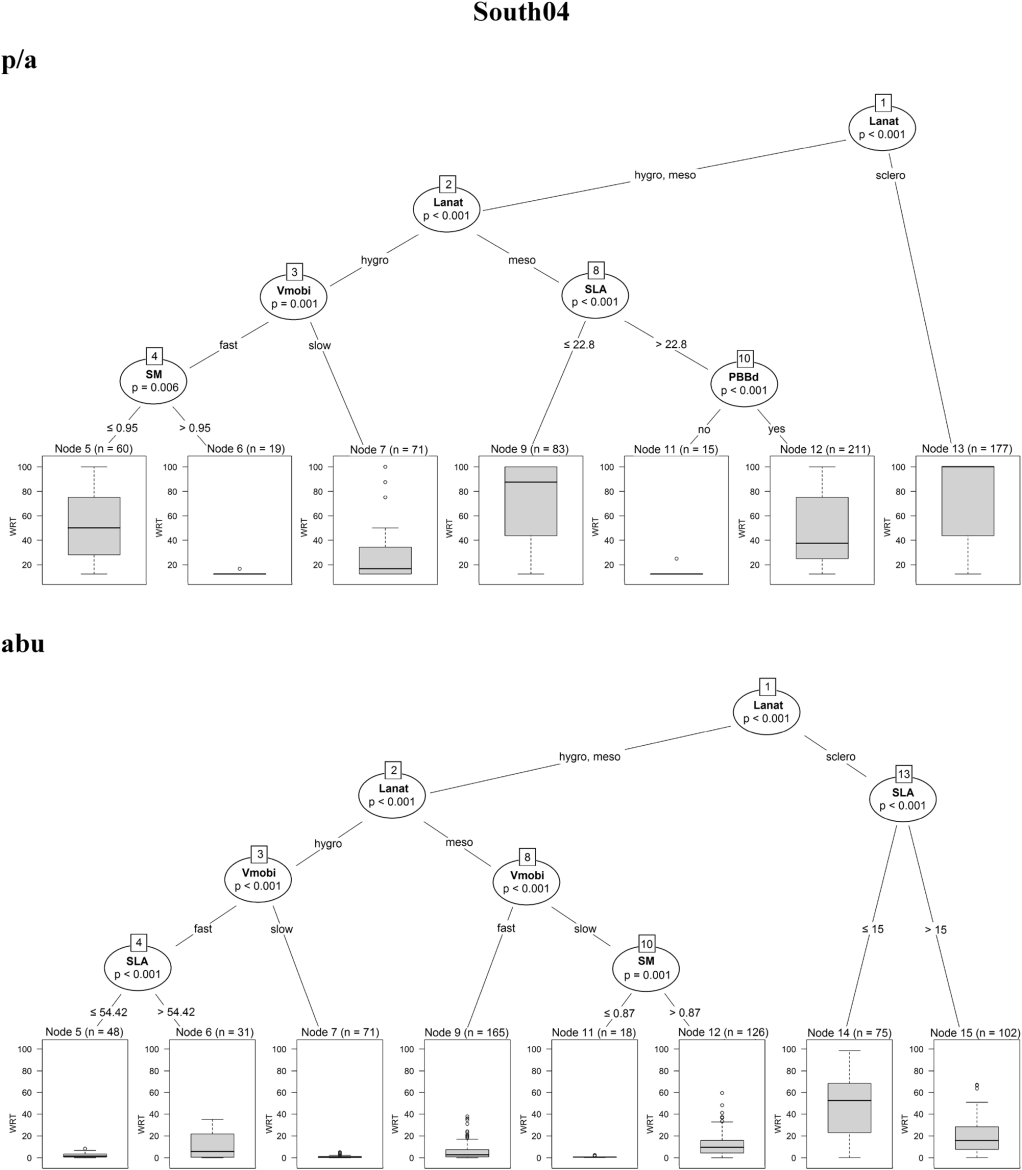
Conditional inference trees for North01, Central02, South03, South04, indicating which plant functional traits are driving species persistence during the time series. Results coming from presence/absence (p/a) and abundance data (abu) are compared. In the split of the trees, all species traits are tested and the trait that best discriminates between homogeneous groups is selected. The response variable (summarised as boxplots) is the weighted residence time (expressed as WRT_p/a_ and WRT_abu_) along the 8 diachronic observations within the time series of 12 years. The split of the tree is described by the trait used at the split, as well as the permutation-based significance of the split (*p*-value) and the trait values at which the split occurs. *Lanat* leaf anatomy, *Helo* helomorphic leaves, *hygro* hygromorphic leaves, *Meso* mesomorphic leaves, *Sclero* scleromorphic leaves, *SM* seed mass, *SLA* specific leaf area, *Vmobi* vegetative mobility, *PBBd* perennial bud bank belowground.

The regression trees for the four sites showed that the selected traits were significantly correlated with the fine-scale species persistence over time. In North01, species with *helomorphic leaves* were solidly persistent when using both presence/absence and abundance data. In addition, by using presence/absence data, the regression tree for North01 was more complex: species with *hygro/mesomorphic leaves* with *higher seed mass* (> 19.7 mg) or *higher SLA* (> 28.8 mm^2^.mg^−1^) and *fast vegetative mobility* were also persistent. On the contrary, species with *lower seed mass* (≤ 19.7 mg) and *lower SLA* (≤ 28.8 mm^2^.mg^−1^) were characterised by the lowest value of persistence (Fig. 2a).

In Central02, both methods showed that the only trait driving species persistence was *seed mass* (Fig. 2b). The most persistent species were those characterised by *higher seed mass* (> 6.9 mg).

In South03, species persistence was mainly correlated with clonal and bud-bank traits (*perennial belowground bud bank* and *vegetative mobility*) (Fig. 2c). In detail, when using presence/absence data, species lacking *perennial belowground bud bank* did not persist. Species with *perennial belowground bud bank, fast vegetative mobility* and a *seed mass* 6.2 < × ≤ 7.1 mg were solidly persistent. In case of *slow vegetative mobility*, only species with *lower SLA* (≤ 24.9 mm^2^.mg^−1^) were moderately persistent. When using abundance data, species with *fast vegetative mobility*, *mesomorphic leaves* and a *seed mass* 3.4 < × ≤ 7.1 mg were solidly persistent.

In South04, the traits driving species persistence were mainly *leaf anatomy, specific leaf area* and *vegetative mobility* by using both presence/absence and abundance data (Fig. 2d). In case of presence/absence data, species characterised by *scleromorphic leaves* or *mesomorphic leaves* with *lower SLA* (≤ 22.8 mm^2^.mg^−1^) were solidly persistent, while, species with *hygromorphic leaves, fast vegetative mobility,* and a very *low seed mass* (≤ 0.95 mg) were moderately persistent as well. In case of using species abundances, species characterised by *scleromorphic leaves* and *lower SLA* (≤ 15.0 mm^2^.mg^−1^) were solidly persistent.

## Discussion

Our study suggests that fine-scale species persistence is correlated with different plant functional traits in different local ecological contexts. Substantial differences in functional traits driving fine-scale patterns of species persistence were mainly observed between the two Northern and the two Southern sites, which showed different resource use and reproductive strategies of persistent species. We suggest that these differences might appear to be dependent on local trait adaptation rather than plant phylogenetic history.

The different ecological contexts (i.e. climate, soil, and topography; see Table 1) might be underpinning the differences in species composition among the studied sites. Overall, our dataset included 87 species, only one of which (*Cardamine bulbifera*) occurred in all four sites, and only seven occurred in three out of four sites (e.g. *Galium odoratum*, *Lactuca muralis, Sanicula europaea*). The vast majority of species (65%) belonged exclusively to one of the sites. Since we used trait values from the literature (i.e. fixed attributes for species), the compositional differences among sites are mirrored by differences in trait abundance distribution.

**Table 1:** The main abiotic features of the studied beech sites.

Site	Coordinates	Elevation. (m)	Slope (%)	Aspect	Bedrock and soil type	Bare soil and rocky outcrops (%)	Stand age (yrs)	Mean annual P (mm year^−1^)	Mean annual T (° C)	Summer P–PET ratio (June–July–August)
North01	+ 460326N + 120156E	1100	10	NW	Limestone–Haplic Luvisol	1	135	1900	5	0.91
Central02	+ 415051N + 133523E	1500	30	SW	Limestone–Aluandic Andosol	1.8	125	1300	10	0.52
South03	+ 402558N + 152610E	1175	30	W	Limestone–Eutrosilic Andosol	4.2	115	1250	10	0.27
South04	+ 382538N + 161047E	1100	20	NE	Granites–Haplic Umbrisol	2.5	125	1500	10	0.19

Data from Fabbio et al.^76^, Hijmans et al.^51^ and Trabucco & Zomer^77^.

Our study highlighted that each of the selected traits related to the challenges to plant persistence was important for the fine-scale species persistence observed in at least one site. Higher values of *seed mass* were correlated with species persistence mainly in the Northern sites (North01 and Central02). This trait played a secondary role in South03 and a marginal role in South04. Therefore, the hypothesis H1 about the general importance of this trait has been rejected. Graae & Sunde^9^ and Aubin et al.^27^ found that *seed mass* is a fundamental trait for the understory layer, with species characterised by heavy seeds being more abundant in ancient forests. Several studies demonstrated that heavy seeds: (i) enable seedlings to tolerate shade by providing a more significant initial energy reserve, and (ii) provide seedlings with the increased height relative to small-seeded species, which can be of an advantage for germination below litter^35,36^. Our results confirmed these findings mainly for the stands less impacted by summer drought. In summary, *seed mass* played a crucial role in Central02 (as indicated by both presence/absence and abundance data), where the particular light regime due to the highest density and lowest height of trees (Table 2) can favour species with heavy seeds. Corroborating this pattern, Central02 showed the lowest values of understory species cover and density^37^.

**Table 2:** The main stand structural features of the studied beech sites. Data from Fabbio et al.^76^.

Site	Tree density (n ha^−1^)	Tree species (No.)	Mean dbh (cm)	Basal area (m^2^)	Mean height (m)	Top height (m)	Canopy depth (m)	LAI (m^2^/m^2^)	Leaf litter (Mg ha^−1^)
North01	345	1	35.8	34.64	23.90	25.20	7.40	5.25	2.318
Central02	899	1	23.7	40.09	19.50	24.60	9.30	4.67	2.969
South03	228	2	51.5	47.57	26.80	28.00	14.10	n.a	2.295
South04	333	2	39.1	39.90	24.10	28.60	14.10	4.36	4.644

Leaf traits demonstrated to be correlated with species persistence mainly at both extremes of the climatic gradient. This pattern is confirmed by both presence/absence and abundance data. Interestingly, in both sites, *leaf anatomy* seemed to be an essential trait in determining species persistence at a fine scale, but with opposite attributes according to the climatic context. This North *vs* South pattern in *leaf anatomy* (i.e. *helomorphic* vs *scleromorphic leaves*, respectively) seems to be consistent with our findings of *higher SLA* of persistent species in the northernmost site and a *lower SLA* of persistent species in South03. These results confirm H2 addressing the importance of *lower SLA* and *scleromorphic leaves* in the southern sites characterised by stronger summer drought. The latter leaf traits are related to water-use strategy and photosynthetic performance^22^. As reported by Wellstein et al.^29^, lower levels of SLA enhance water use efficiency in arid environments, as shown for the Mediterranean vegetation^38,39^. On the contrary, higher levels of SLA and leaf water content led to higher relative growth rates. The significance of such traits in determining species persistence in forest ecosystems was also confirmed by other studies based on more extended time (20 to 50 years^40,41^) or a long chronosequence (90 years^14^).

Clonal and bud-bank traits were correlated with species persistence at fine scale mainly in the Southern stands characterised by stronger summer drought (South03, South04), confirming the H3 that clonal and bud-bank traits are crucial for species persistence in forests more impacted by summer drought. Clonality increases fine-scale spatial resource acquisition, assisting plants to persist under stressful conditions^15,33,42^. Further, having a perennial bud-bank is related to the capacity of regeneration after disturbance^43^. In fact, they are mainly important in determining species persistence at a fine scale in South03 which is characterised by a combination of steep slopes and rocky soil (Table 1). This site is embedded in pasture-dominated landscape^41^ where the potential impact of grazing can likely affect the forest understory vegetation. Several studies found clonal and bud-bank traits to be important in disturbed forests, that is, post-harvested and grazed stands^34,41^, but also in more water stress-prone mature forest stands characterised by steep slopes with diminished soil depth, fertility and moisture^14,15^. In support of our findings, Campetella et al.^14^ found a strong correlation between stand slope and the occurrence of a large and perennial bud bank.

Interestingly, the most important traits determining fine-scale species persistence were generally consistent when using presence/absence and abundance data (i.e. Leaf anatomy in North01 and South04; Seed mass in Central02). This result reveals a similar trait-driven persistence behaviour of both dominant and subordinate species. Only in South03 the most important trait correlated with species persistence changes according to the used approach: with presence/absence data, perennial bud bank belowground was hierarchically first and vegetative mobility second. On the contrary, with abundance data, vegetative mobility was hierarchically first and perennial bud bank belowground third. However, these traits reflect the same plant strategy: being clonal and having a bud bank, provides plants with an effective strategy to cope with disturbances and changing environments^44^.

Some differences were detected between the two approaches (i.e. using presence/absence or abundance data) in terms of structure of the regression trees, number of significant traits, and their relative importance. Particularly, the differences appear in sites characterised by higher values of understory species cover and density^37^. On the contrary, they were perfectly consistent in Central02, which is featured by the lowest species cover and density^37^. This clearly reflects the sensitivity of the approach with respect to the community structure. The abundance method properly accounts for aboveground species dominance, thus recognizing the proportionally greater effect of dominant species on the ecosystem properties with respect to subordinate species^45^. However, the combined use of both methods can reveal different persistence strategies of dominant *vs* subordinate species in communities with low values of evenness.

Finally, we suggest that the same species abundance value can be reached through different strategies of space occupation (e.g. single and not interconnected ramets, dense tussocks, interconnected ramets with above- or belowground connections). In some species, these strategies can change over time as an adaptive response to environmental conditions^46^. This aspect can be extremely relevant for plant persistence at fine scale and should deserve particular attention^47^.

Our study, using permanent monitoring plots in four old-growth beech forest stands, demonstrated how fine-scale species persistence is correlated with different functional strategies, depending on local ecological factors. Local functional strategies were found not to be related with phylogenetic history, but seem to be dependent on trait adaptation. It suggests that, in these old-growth forests characterised by relatively stable ecological conditions (e.g. forest structure and canopy closure), understory dynamics are primarily governed by the fit of species to their abiotic environment (ecological filter)^48^. The maintenance of ecological continuity is crucial for the persistence, and consequently, for the conservation, of specialist species founding niche in the understory of old-growth forests.

Persistent species in stands characterised by less marked summer water stress showed an acquisitive resource use strategy (helomorphic leaves with high SLA) and higher seed mass. In contrast, in the old-growth beech forests with potential summer soil water depletion, persistent species seemed to adopt a conservative resource-use strategy (scleromorphic leaves with low SLA), supported by clonal and resprouting abilities. We assessed the species persistence through survival of the aboveground organs. Naturally, the disappearance of aboveground organs does not mean the disappearance of a plant individual as it may survive the form of belowground organs. In addition, we acknowledge that the patterns reported in the present study do not account for intraspecific trait variation. Future research should (a) explore the belowground dimension of plant persistence, hence of the persistence of belowground organs, (b) assess the interactions between plant persistence and resident microbial community, and (c) incorporation of intraspecific trait variability into the notion of persistence since the trait variability may play a fundamental role in plant community responses to the environment^49^.

## Materials and methods

### Study area

We selected four sites of the Italian forest ecosystem monitoring network (CONECOFOR^50^) located in old-growth (> 100 years old) beech forests, spanning a latitude of 46°03′ N–38°25′ N, with mean annual precipitation of 1900–1250 mm. The sites are located in the following Italian political regions: Veneto (North01), Abruzzo (Central02), Campania (South03), and Calabria (South04) (Fig. 1; Table 1). Fifty years^51^ of monthly temperature and precipitation data^52^ indicate that the North01 and Central02 sites do not experience aridity in summer, while the Southern sites (South03 and South04) are exposed to summer drought (Fig. 1). Analysis of the ratio between Precipitation and Potential Evapotranspiration (P/PET) in the summer months^53^ (June- to August) indicates that the North01 site experiences a slight potential summer drought (0.92), while the Southern sites (South03 and South04) show marked potential summer drought (0.27 and 0.19, respectively; Table 1). According to the beech distribution^54^, South04 exemplifies one of the southernmost beech forests in Europe.

### Sampling design

Within each 50 × 50 m site, we established a systematic grid of non-contiguous permanent sampling units (0.50 m × 0.50 m each; Appendix S1), separated by at least 5 m. This minimum distance assured that spatial autocorrelation would be rendered non-significant (see Appendix S2). In each sampling unit, presence/absence and abundance (species cover values, %) of the herb layer vascular plant species were recorded over twelve years (1999–2011), during eight surveys (1999, 2000, 2001, 2002, 2005, 2006, 2008, and 2011; Appendix S3). The data collection protocol followed the ICP Forests Manual on Ground Vegetation Assessment^55^ in order to ensure dataset commensurability^56^. The sampling was always performed during the optimal growing period in the pertinent regions.

### Plant functional traits

Among the different traits related to species persistence^22^, we selected (i) not correlated traits, and (ii) traits with available attributes to cover our list of species (see Appendix S4 for details). This selection includes five above- and belowground plant traits (Table 3), such as (1) *seed mass*, pertinent to space/time dispersal ability and seedling establishment; (2) *perennial belowground bud bank,* reflecting resprouting ability after disturbance; (3) *specific leaf area* (*SLA*), related to water use strategy and photosynthetic capacity), (4) *leaf anatomy*^27^, related to leaf water and nutrient conservation and prevention of mechanical damage^31^; and, (5) *vegetative mobility*, pertinent to fine-scale spatial resource acquisition^11,32,33^. The traits mentioned above are likely to be useful for predicting plant responses to disturbance and a broad range of environmental factors^22^. Traits values were obtained from existing database and literature sources^14,30,57–61^ and partly from our field measurements using international standardised sampling measurement protocols^62^. Information was available for 96% of the studied species. We did not consider intraspecific trait variability and used fixed trait values for each species.

**Table 3:** Summary of the selected plant functional traits. For detailed on the trait selection process see Appendix S4.

Plant trait	Description	Data type	Data source
Seed mass	Oven-dry mass of an average seed of a species	Quantitative	^14,58,59^
Perennial bud bank (belowground)	Bud bearing organs persisting for 2 or more years are classified as perennial	Binary	^14,57^
Specific leaf area	One sided area of a fresh leaf divided by its oven-dry mass	Quantitative	^14,60,61^
Vegetative mobility	Lateral spread per year	Categorical	^14,57^
Leaf anatomy	Leaf structure according to water content and gas exchange	Categorical	^30^

### Data analyses

#### Differences in species composition and trait abundance among the sites

To ascertain whether species composition and trait abundance significantly vary between the four selected sites at the start of the sampling period, we tested for the significance of compositional change with a permutational analysis of variance (PERMANOVA) based on the Bray–Curtis dissimilarity index^63^. The PERMANOVA test performs a permutational test (999 permutations) using distance matrices of the species and traits abundance composition, to find significant differences between sites^64^.

### Relationship between functional and phylogenetic patterns

To assess if the trait patterns depended solely on local trait adaptation or if potential evolutionary signal reflected by phylogenetic patterns could be detected, we compared functional and phylogenetic diversity at plot level in each site, using the following approach:

1. The evolutionary history of the species in our dataset was inferred using the Neighbor-Joining method^65^. The evolutionary distances were computed using the Maximum Composite Likelihood method^66^ and are in the units of the number of base substitutions per site. This analysis involved 62 nucleotide sequences. All ambiguous positions were removed for each sequence pair (pairwise deletion option). There were a total of 2145 positions in the final dataset. Evolutionary analyses were conducted in MEGA X^67^. In case of missing species we used similar species of the same genus. (see Appendix S5 for the phylogenetic tree).
2. We then calculated both functional (FD) and phylogenetic diversity (PD) for each 0.5 × 0.5 m plot in each site for the first year (i.e. 1999) by using the mean pairwise trait distances based on Gower distance^68^. Specifically, FD has been calculated using a set of numerical and categorical traits listed in the manuscript.
3. The FD and PD scores at plot level were correlated in each site (through Spearman's rho correlations) in order to explore if there is a relationship between the two diversity indices.

#### Species persistence assessment

Different methods have been proposed to assess species persistence, including those based on immigration/extinction rates or other indirect approaches (for instance involving differences with respect to randomly generated patterns)^5^. We evaluated species persistence starting from the concept of local aboveground survival (i.e. recording directly whether a species remains present aboveground during specific years or a specified interval of observation). We introduced a new index, based on the estimation of the *weighted residence time*, or mean residence time, that is, the mean period in which a species is observed in the same sampling unit over the surveyed time. We calculated the *weighted residence time* in each sampling unit of each site using the following formula:

$$WRT = { 1}00{\text{ x }}\left( {{\text{P}}_{im} /{\text{ T}}_{im} } \right)/{\text{Z}}$$

where *WRT* is the persistence as *weighted residence time*, P_*im*_ is the sum of occurrences of species *i* in sampling unit *m* over the surveyed period, T_*im*_ are the non-consecutive occurrences of species *i* in sampling unit *m* over the surveyed period, and Z is the total number of surveys. Persistence values can be based on both species presence/absence and abundance data (see Table 4 for details).

**Table 4:** Concepts for species persistence estimation with the proposed “Weighted residence time” (WRT) method for different species (sp_n_) over time (t_n_), by using both presence/absence (p/a) and abundance data (abu). To obtain WRT with abundance data, we weighted WRT_(p/a)_ with respect to the mean relative species cover (Rel.cov.) over time (i.e. WRT_(abu)_ = WRT_(p/a)_*Rel.cov.).

	t_1_	t_2_	t_3_	t_4_	t_5_	t_6_	t_7_	t_8_	WRT_(p/a)_	Rel.cov	WRT_(abu)_
sp_1_	1	0	1	0	1	0	1	0	12.5	0.5	6.25
sp_2_	1	1	0	0	0	0	0	0	25	0.6	15
sp_3_	0	0	1	1	0	0	1	0	18.6	0.3	5.58
sp_4_	0	0	1	1	1	0	1	0	25	0.4	10
sp_5_	1	1	1	1	1	1	1	1	100	0.7	70
sp_6_	1	1	1	1	1	1	1	0	87.5	0.8	70
sp_7_	1	1	1	0	1	1	0	0	31.3	0.5	18.7

#### Species persistence related to plant functional traits

To evaluate whether specific traits are correlated with the patterns of species persistence at a fine scale in all the successive periods (i.e. 1999‒2000; 2000‒2001; 2001‒2002, etc.), we constructed conditional inference trees (CITs) based on the relationship between *WRT* (both with presence/absence and abundance data) of species estimated within each of the 100 quadrates in the grid, and the list of selected traits. A conditional inference tree (also known as classification and regression tree, CART^69,70^) estimates whether the selected traits determine species partitioning into homogeneous groups concerning changes in species occurrence between the sampling events^40^. The algorithm selects the input variable with the strongest association with the response, and it stops if the null hypothesis of independence between the input variables and the response cannot be rejected. We used CITs because they (a) account for nonlinear hierarchical relationships, (b) treat categorical, ordinal and quantitative data simultaneously, and (c) deal with missing values^69,71^. In each split of the tree, all species traits are tested, and the trait that best discriminates between homogeneous groups of species is selected.

All statistical analyses were performed in the R environment, on the incidence matrix with species presence/absence and abundance data. In particular, the following R packages were used: package vegan^72^ (function *adonis*) for PERMANOVA, package party (function *ctree*) for regression trees^73^, package picante^74^ for the calculation of FD and PD, package FD^75^ for the Gower distance matrices of traits.

## Supplementary information

Supplementary Information
