# Supplementary material for: Plant functional traits are correlated with species persistence in the herb layer of old-growth beech forests
Source: Sci Rep. 2020 Nov 6;10:19253. doi: 10.1038/s41598-020-76289-7 (PMC7648635; doi:10.1038/s41598-020-76289-7)

**Plant functional traits are correlated with species persistence in the herb layer of old-growth beech forests**

Giandiego Campetella^1^, Stefano Chelli^1*^, Enrico Simonetti^1^, Claudia Damiani^2^, Sandor Bartha^3,4^, Camilla Wellstein^5^, Daniele Giorgini^1^, Nicola Puletti^6^, Ladislav Mucina^7,8^, Marco Cervellini^1,9^ & Roberto Canullo^1^

^1^ Unit of Plant Diversity and Ecosystems Management, School of Biosciences and Veterinary Medicine, University of Camerino, Via Pontoni, 5, I-62032 Camerino, Italy.

^2^ School of Biosciences and Veterinary Medicine, University of Camerino, Via Gentile III da Varano, I-62032 Camerino, Italy.

^3^ Institute of Ecology and Botany, Centre for Ecological Research, H-2163 Vácrátót, Hungary.

^4^ GINOP Sustainable Ecosystems Group, Centre for Ecological Research, Klebelsberg Kuno u. 3, H-8237 Tihany, Hungary.

^5^ Faculty of Science and Technology, Free University of Bozen-Bolzano, Piazza Università 5, I-39100 Bozen, Italy.

^6^ Council for Agricultural Research and Agricultural Economy Analysis, Forestry Research Centre, Viale Santa Margherita 80, I-52100 Arezzo, Italy.

^7^ Harry Butler Institute, Murdoch University, 90 South Street, Murdoch WA 6150, Perth, Australia.

^8^ Department of Geography and Environmental Studies, Stellenbosch University, Private Bag X1, Matieland 7602, Stellenbosch, South Africa.

^9^ Department of Biological, Geological, and Environmental Sciences, Alma Mater Studiorum, University of Bologna, I-40126 Bologna, Italy.

* Corresponding author: Stefano Chelli, stefano.chelli@unicam.it

**SUPPLEMENTARY**

**Appendix S1.** A scheme of the sampling design adopted for vegetation monitoring of the CONECOFOR plots. The main forest stand is divided into twenty-five 10 m x 10 m sections; 100 quadrats 0.50 m x 0.50 m were set up for the monitoring at the population level (black squares along diagonals).

**Appendix S2.** Moran I spatial autocorrelation error according to Legendre & Legendre (1998) . Three distances (5 m, 10 m, 15 m) among micro-plots were considered, and both plant cover and species combination were tested. All errors due to spatial autocorrelation are not significant (critical value for multiple test *p* < 0.01).

| PMP | Distance | Plant cover | Species combination |
| --- | --- | --- | --- |
| North01 | 5 m | -65.88 _NS_ | -1.15 _NS_ |
|  | 10 m | -55.89 _NS_ | 11.28 _NS_ |
|  | 15 m | -24.55 _NS_ | 1.56 _NS_ |
| Central02 | 5 m | -746.56 _NS_ | 1.41 _NS_ |
|  | 10 m | -95.08 _NS_ | -1.81 _NS_ |
|  | 15 m | -64.66 _NS_ | 0.46 _NS_ |
| South03 | 5 m | 1228.10 _NS_ | 69.55 _NS_ |
|  | 10 m | 673.28 _NS_ | 38.05 _NS_ |
|  | 15 m | -113.76 _NS_ | 12.51 _NS_ |
| South04 | 5 m | 557.76 _NS_ | 153.48 _NS_ |
|  | 10 m | 276.89 _NS_ | 73.39 _NS_ |
|  | 15 m | 326.90 _NS_ | 52.93 _NS_ |

**Reference**

Legendre P., & Legendre L. Numerical ecology, second English Edition. Elsevier, 853 pp. (1998).

**Appendix S3.** Tables with plant species list, families, life history syndromes and occurrence in sampling units per year.

NORTH01

CENTRAL02

SOUTH03

SOUTH04

**Appendix S4. (a)** List of the PFTs related to challenges to species persistence (according to Weiher et al. 1999). **(b)** Correlation among the five selected PFTs: the table shows the Spearman's Rho values and the significance level (NS = not significant); the selected PFTs are not significantly correlated.

**(a)**

Seed mass

Dispersal mode

Seed shape

Specific leaf area

Leaf water content

Height

Life history

Stem density

Clonality (traits related to vegetative mobility)

Resprouting ability (traits related to bud bank)

From this list, we selected (i) not correlated traits, (ii) the ones with more information available in the literature and databases and (iii) the ones covering more detailed attributes (i.e. instead of ‘occurrence of clonal growth’ we selected ‘vegetative mobility’).

(b)

| **Spearman's rho** | Perennial bud bank (below-ground) | Specific leaf area | Vegetative mobility | Leaf anatomy |
| --- | --- | --- | --- | --- |
| Seed mass | 0.150 NS | -0.062 NS | 0.046 NS | -0.008 NS |
| Perennial bud bank (below-ground) |  | -0.184 NS | 0.130 NS | 0.125 NS |
| Specific leaf area |  |  | 0.113 NS | -0.197 NS |
| Vegetative mobility |  |  |  | -0.033 NS |

**Appendix S5.** Phylogenetic tree of the species occurring in our dataset. The evolutionary history of the species was inferred using the Neighbor-Joining method. The optimal tree with the sum of branch length = 11.07950685 is shown below. The tree is drawn to scale, with branch lengths in the same units as those of the evolutionary distances used to infer the phylogenetic tree. In case of missing species we used similar species of the same genus.


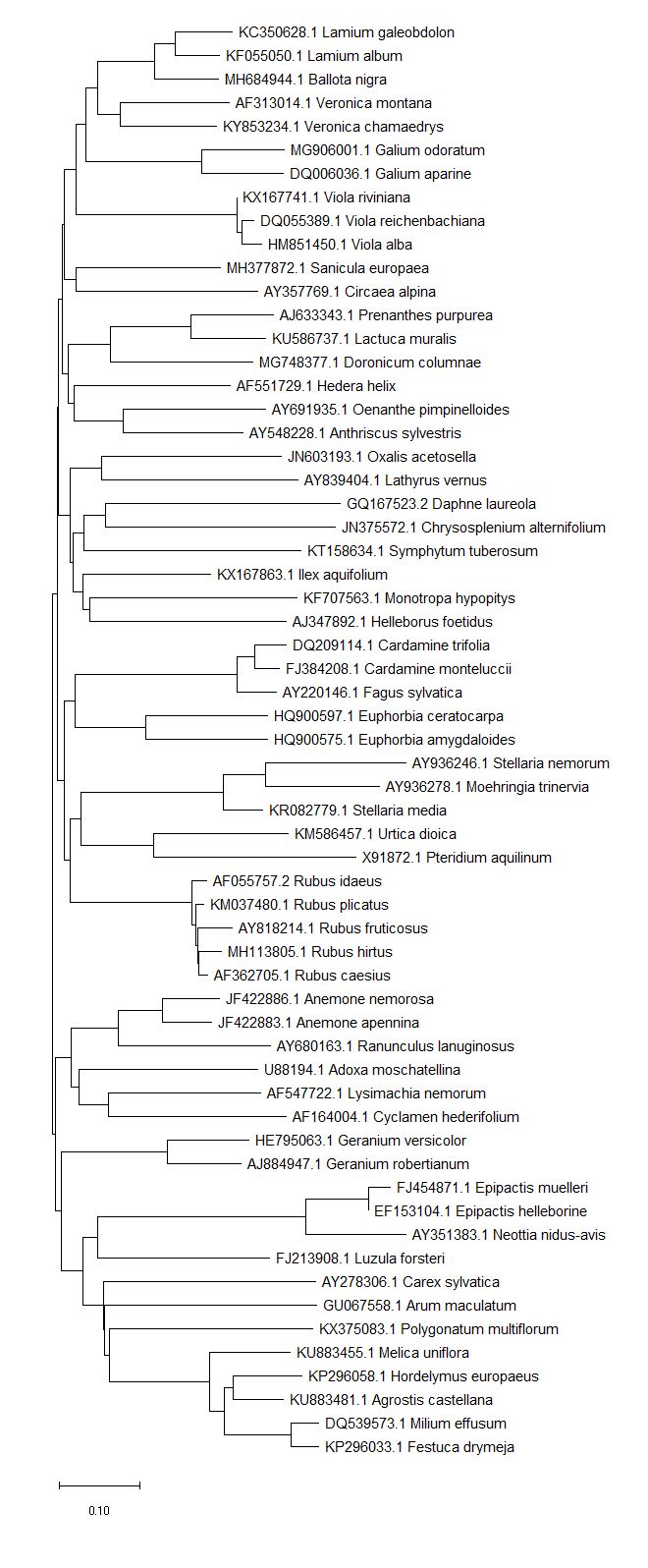

Supplement: Supplementary file 1 — Supplementary Information [file 41598_2020_76289_MOESM1_ESM.doc]
